# The relationship between health-related knowledge and attitudes and health risk behaviours among Portuguese university students

**DOI:** 10.1177/17579759231195561

**Published:** 2023-09-16

**Authors:** Regina F. Alves

**Affiliations:** CIEC – Research Centre Child Studies, Institute of Education – University of Minho, Braga, Portugal

**Keywords:** health risk behaviour, health risk knowledge, attitudes towards health, higher education, health education

## Abstract

Scientific evidence reveals a high prevalence of health risk behaviour among university students. This calls for the creation of educational programmes that promote more knowledge about health. However, knowledge alone is not enough to change behaviours; other factors should be considered, including attitudes towards health. The objective of this cross-sectional study was to analyse the relationship between knowledge, attitudes and health risk behaviours among university students. For this, a previously validated self-report questionnaire was applied to a stratified sample of 840 students, by year of study (first- and third-year students) and their scientific area. In addition to sociodemographic issues, the questionnaire contains a health-related knowledge scale, an attitudes towards health scale, and questions about health risk behaviours. Students displayed poor knowledge about health, correctly answering 17.77 (SD = 4.59) questions out of a total of 36, and moderate scores concerning attitudes towards health (*M* = 2.61, SD = 0.48, range: 1–5). Students reported always engaging in, on average, 3.88 (SD = 1.45) of the seven behaviours subject to the analysis. Mediation analyses indicated that knowledge about health and attitudes towards health were statistically significant predictors of risky behaviours. Furthermore, it was indicated that attitudes towards health have a mediating effect between health knowledge and health risk behaviours. Findings from this study indicate that public health and education policies should promote healthy behaviours among university students, taking into account not only the level of knowledge but essentially the development of positive attitudes when facing behaviours which put health at risk.

## Introduction

Scientific research examining the health of university students has consistently shown that they are more likely to engage in risky behaviours such as excessive alcohol consumption, tobacco and drug use, physical inactivity, unbalanced eating habits and risky sexual behaviour (1–3). In addition, studies have indicated a correlation between multiple risk behaviours and a higher prevalence among final-year students compared with first-year students (4). Possible factors contributing to this trend include curiosity, experimentation, reduced parental control, identity development and the normalisation of these behaviours in the academic environment. The persistence of these unhealthy behaviours acquired during higher education has significant implications for long-term health and well-being, highlighting the urgent need for research in this field (5–7).

Health risk behaviours are a significant issue among university students and are commonly linked to various health problems, including chronic non-communicable diseases (8). In addition, these behaviours are associated with academic challenges (e.g. low academic engagement and performance), psychosocial and legal problems (e.g. decreased ability to concentrate, sexual assault, drug overdose, memory impairment, persistent cognitive deficits, increased risk of suicide, risky sexual practices, property damage, driving under the influence of alcohol, injuries, violence and traffic accidents) (9,10).

Health behaviour is influenced by various factors, including knowledge and attitudes. These factors have been identified in different research theories within the field of health education, with one notable model being the rational knowledge attitude practice (KAP) model. The KAP model assesses individuals’ knowledge about health information, their attitudes towards a specific issue, and the practices they adopt. Some studies have also incorporated individuals’ beliefs, particularly misconceptions that hinder appropriate behaviour (11). Thus, in the realm of health behaviour studies, KAP surveys primarily serve to gather data on individuals’ understanding, beliefs and actions concerning a particular issue. These surveys aid in the planning, implementation and evaluation of programmes, as well as in identifying gaps in knowledge, cultural beliefs, or behavioural patterns that may either facilitate or hinder the success of a programme (12).

Although the relationship between knowledge, attitudes and health behaviours among university students continues to be extensively researched, most studies have focused on specific health behaviours (13–15). Research has shown that students often have low levels of health-related knowledge (15–17), suggesting that increasing knowledge about the dangers of risky behaviours could reduce the likelihood of engaging in such behaviours (18). However, while knowledge is essential to promoting healthy behaviours (19), it is not sufficient on its own. Therefore, effective interventions should not only provide information about health risks and hazards but also equip people with the necessary skills to avoid them (20). In this framework, attitudes play a crucial role in predicting health risk behaviours (21) and can facilitate the adoption of healthy behaviours. For example, positive attitudes towards contraceptive methods may increase the likelihood of condom use to prevent sexually transmitted infections (STIs) (22), while negative expectations (such as impaired cognitive abilities) may lead to reduced alcohol consumption (23). Nevertheless, knowledge is essential to developing attitudes towards different behaviours and helping individuals make decisions and choose healthy behaviours (24).

### The present study

The above literature provides a basic understanding of the KAP framework. In this context, the present study aims to determine the relationship between knowledge, attitudes and health risk behaviours among university students and how these factors are interrelated. In this study, a wide range of health risk behaviours (smoking, alcohol consumption, illicit drug use and self-medication, eating habits, physical activity and sexual risk behaviours) are analysed. A mediation model analysis is used to test the hypotheses developed based on the KAP model: H1: there is a relationship between knowledge level and health risk behaviours; H2: attitudes towards health affect the prevalence of risk behaviours; H3: health knowledge impacts attitudes towards health; and H4: attitudes towards health have a mediating effect between health knowledge and risk behaviours. These hypotheses were also tested separately for each of the behaviours.

## Materials and methods

### Research method

A cross-sectional study was conducted with a proportionally representative sample of Portuguese university students at a public university during the 2018–2019 academic year.

### Participants and sample

The population studied included first- and third-year bachelor’s and master’s degree students (*N* = 5447). A stratified sample was then formed by the year of study (first and third) and field of study (human and social sciences, law and economic sciences, exact and natural sciences, and engineering sciences) to ensure proportional representativeness of the target population. Thus, classes related to health, master’s or postgraduate courses, courses that did not have classes in the first and third years, and evening classes were excluded. The minimum sample size needed for this study was 592 students (margin of error = 5%, confidence level = 99% and response distribution = 50%). A total of 873 university students were invited to participate, and 33 questionnaires were excluded because they had not been answered or filled out incorrectly. The response rate was 96.2%.

The total number of participants was 840 university students, mainly female (*n* *=* 465, 55.4%) and those attending university full-time (*n* = 739, 88.8%). On average, students were 20.78 (SD = 4.221) years old. Consistent with the make-up of the student body, most respondents attended the first year of study (*n* = 464, 55.2%), and a large proportion was in the scientific engineering field (*n* = 302, 36.0%).

### Instruments

Following the objectives of the study, the scales of this study were developed in three stages: construction of the scale (Stage 1), content validity (Stage 2) and psychometric validity (Stage 3), as set out by the World Health Organization’s (WHO’s) guide to developing KAP surveys (25). The scales were developed based on a literature review identifying existing instruments for measuring KAP. The items commonly used to assess knowledge and attitudes (perceptions, beliefs and intentions regarding health behaviours) were compiled and subjected to a pilot test involving five national scientific researchers and 12 undergraduate students. This final version was then administered to a separate group of 32 students.

### Health risk behaviour

This scale was divided into seven categories. Smoking status was determined by the question ‘Do you currently smoke?’ and analysed according to the classification: non-smoker, former smoker or current smoker.

Alcohol consumption was measured using the AUDIT-3 scale (three items with a five-point scale coded from 0 to 4, range 0–12, with risky alcohol consumption identified in males from scores of 4 and females from scores of 3) (26).

The consumption of illicit drugs included three questions about the use of cannabis, cocaine and hallucinogens (‘In the past 12 months, how many times have you consumed. . .?’). The consumption of illicit drugs was classified as a binary item: use of at least one of the listed psychoactive substances.

The prevalence of self-medication was measured by the question, ‘In the last 12 months, how many times have you consumed any of the psychoactive substances listed: antidepressants/sedatives/relaxants/tranquilisers (without prescription); analgesics/anti-inflammatories (without prescription); vitamins/nutritional supplements (without prescription)?’ Self-medication was classified as such if one of the psychoactive substances had been used without a prescription at least once in the previous year.

Healthy dietary habits were assessed using four food groups over the past seven days, including vegetables, fruit, sweets and fast food. Students were also asked how often they had skipped breakfast, lunch and/or dinner in the past seven days. For the analyses, unhealthy eating habits were considered one of the following behaviours in the last seven days: skipping breakfast, skipping lunch and/or dinner, insufficient fruit consumption (two or fewer times per day), insufficient consumption of vegetables (two or fewer times per day), higher consumption of sweets (four or more times per week) and higher fast food consumption (four or more times per week), according to the WHO’s guidelines for a healthy diet (27).

The Godin leisure-time exercise questionnaire (28) was used to measure the prevalence of physical activity. Total activity scores were calculated by adding the metabolic equivalent calculations for each physical activity intensity level (multiplying episodes of vigorous activity by 9, moderate activity by 6 and mild activity by 3), and fewer than 14 units were classified as sedentary.

Sexual risk behaviours were measured with five questions, where risky sexual behaviours were considered to be at least one of the following: first sexual intercourse at the age of 16 or younger, two or more sexual partners in the past 12 months, failing to use a condom in all sexual relations in the past 12 months and sexual relations after alcohol or drug use in the past 12 months.

The health risk behaviour variable was calculated by summing the answers to each category, ranging from 0 to 7, with the highest value corresponding to the higher number of health-related risk behaviours. In addition, each behaviour was analysed by the dichotomous variable of the presence or absence of this risky behaviour, and it was defined as a positive behaviour if students reported up to two risk behaviours and a negative behaviour if they reported three or more.

### Health-related knowledge

This scale comprised 36 items: six each on tobacco, alcohol, nutrition, sexuality and physical activity and three each on illicit drugs and medication. The following options were available for answering the questions on the scale: ‘true’, ‘false’ and ‘I don’t know’. One point was given for each correct answer, while an incorrect answer or the ‘I don’t know’ response earned 0 points. The sum of all items was calculated, and the scores ranged from 0 to 36; thus, higher scores corresponded to a higher level of knowledge. Poor knowledge was determined when the overall marks were less than 50% of the total score, while a score >50% was considered good knowledge. This scale showed good internal consistency (Cronbach’s alpha (α) = 0.828).

### Attitudes towards health

This scale consisted of 30 items on a five-point Likert scale (1: ‘strongly disagree’, 5: ‘strongly agree’), which included attitudes towards the health-related behaviours listed in the health risk behaviour scale. The scale had five items for each studied behaviour: ‘smoking helps to relax and reduce stress’; ‘a party is more fun when alcoholic beverages are consumed’; and ‘young people try drugs due to emotional problems’. The results of this scale showed the following dynamics: the higher the average of the scale, the more negative the university students’ attitudes towards health were, ranging from 1 to 5. Positive attitudes were found when the total marks were less than 50% of the total score, while >50% was considered a negative attitude. The Cronbach’s α was 0.769.

### Procedure and statistical analysis

After explaining the study’s objectives and receiving informed consent, the researcher gathered the data in a classroom. The paper-and-pencil questionnaire took about 20 minutes to complete.

Data were analysed using IBM SPSS Statistics for Windows, version 26.0 (IBM Corp., Armonk, New York, USA) and PROCESS (version 3.5, 2018).

The descriptive analyses are presented in absolute (*n*) and relative (%) frequencies, means (*M*) and standard deviations (SD). The Pearson test was used to evaluate the correlation between the study variables at a significance level of ⩽0.05. Cronbach’s α was used to assess reliability, while the Shapiro–Wilk test was used to evaluate the normal distribution. A Hayes bootstrapping mediation model (29) with 5000 samples was used to analyse the mediating role of attitudes concerning health, using the designation ‘Model 4’ and a 95% confidence interval (CI).

This analysis calculated the following five paths: the *a* path links the predictor variable for health-related knowledge to the predictor variable for attitude to health (H3); the *b* path links the mediating variable for attitude to health to the outcome for health risk behaviours (H2); the *c* path refers to the overall effect (of the predictor variable for health-related knowledge for the health risk behaviours outcome, without adjustment for the mediating variable for risk) (H1); the direct effect for the *c* path (of the predictor variable for health-related knowledge for the health risk behaviours outcome variable in the presence of the mediating variable attitudes towards health) (H4); and the indirect effect (calculated as the product of measuring the a*b paths) (29).

## Results

### Descriptive analyses

According to the findings, 20.1% of the students surveyed were smokers; 22.2% used illicit drugs; and more than half (60.1%) were at risk of alcohol consumption, while 54.3% had used some form of self-medication in the past year. In addition, more than a third of the students surveyed were inactive (35.7%), 94.2% had an unbalanced diet and 74.9% had engaged in risky sexual behaviour in the previous 12 months. On average, students reported being involved in 3.88 (SD = 1.45) out of the seven behaviours and 82.1% (*n* = 377) engaged in negative behaviours. No significant differences were found in relation to student gender, *t*(457) = 1.013, *p* > 0.05.

As noted in Supplemental material file 1 online, the students’ health knowledge was found to be poor, with a mean score of 17.71 (SD = 4.59) out of 36 questions. More than half of the students had poor knowledge (*n* = 450, 53.6%). The topic of risky sexual practices showed the highest level of knowledge (*M* = 3.99, SD = 1.19), while their knowledge about alcohol scored the lowest (*M* = 2.10, SD = 1.14).

Regarding attitudes towards health, students showed moderate scores (*M* = 2.61, SD = 0.48), with a significant majority (89.4%, *n* = 694) having negative attitudes. The most favourable attitude was observed in the tobacco subscale (*M* = 1.85, SD = 0.79). However, students’ attitudes towards using illicit drugs and alcohol were above the mean of the subscales (*M* *=* 3.66, SD = 0.78; *M* *=* 3.14, SD = 0.84) (Supplemental file 1).

The results of the study showed that health knowledge was positively associated with risk behaviours (*r* = 0.146, *p* = 0.01) and health attitudes (*r* = 0.083, *p* = 0.05), the latter being correlated with behaviours (*r* = 0.293, *p* < 0.01). In addition, statistically significant relationships were observed between all subscales of the health knowledge scale, ranging from 0.162 (*p* < 0.01) to 0.405 (*p* < 0.01), and between all subscales of health attitudes (*r* = 0.081, *p* < 0.05 to *r* = 0.435, *p* < 0.01), except for attitudes towards illicit drugs and self-medication behaviours (Supplemental file 2).

Similarly, knowledge about alcohol, tobacco and illicit drugs was positively associated with risk behaviours (*r* = 0.162, *p* < 0.01; *r* = 0.103, *p* < 0.05; *r* = 0.197, *p* < 0.01, respectively). These results suggest that higher levels of knowledge in these categories increase the likelihood that individuals will engage in health-related behaviours.

### Measurement model

**Figure 1.**
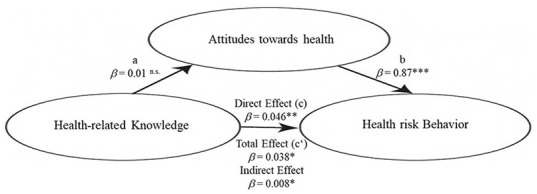
Final structural model. *p < 0.05, **p < 0.01, ***p < 0.001.

This study examined the relationship between health knowledge and health risk behaviours measured by health attitudes. The results showed that health attitudes accounted for 16.7% of the total variance in risk behaviours (*R*^2^ = 0.1358, *F*(1,412) = 7.735, *p* < 0.001). However, H3, which predicted that health knowledge would significantly impact health attitudes, was not empirically supported (β = 0.01, *p* > 0.05). On the other hand, H2, which suggested that health attitudes would significantly predict risk behaviour (β = 0.87, *p* < 0.001), was supported. This indicates that students with negative attitudes are more likely to engage in risky behaviours regardless of their health knowledge. Figure 1 illustrates these results.

Significant results were found for both the direct effect (c, H1; β = 0.046, *p* < 0.01) and the overall effect (c, H4; *β* = 0.038, *p* < 0.05). Furthermore, the indirect effect was 0.008 (95% CI: 0.001–0.017) and statistically significant (*p* < 0.05). These results suggest that students with more health knowledge are more likely to engage in health-related risk behaviours due to their attitudes towards health.

The results of the effect variables for each category analysed in the relationship between knowledge and risk behaviours are presented in Supplemental file 2.

For alcohol, higher knowledge about alcohol had a significant effect on risky consumption patterns (β = 0.095, *p* < 0.001), even after accounting for attitudes towards alcohol consumption (β = 0.060, *p* < 0.001). Similar results were found for illicit drugs and self-medication. Higher knowledge had a significant effect on the consumption of these substances (β = 0.121, *p* < 0.001; β = 0.042, *p* < 0.05, respectively) and attitudes did not cancel out this effect (β = 0.120, *p* < 0.001; β = 0.048, *p* < 0.05, respectively). However, knowledge about tobacco did not affect being an active smoker. Still, negative attitudes towards tobacco use were associated with a higher likelihood of being or becoming an active smoker (β = 0.173, *p* < 0.001). Similarly, knowledge about the benefits of physical activity did not affect a sedentary lifestyle, but positive attitudes towards physical activity positively affected actual physical activity (β = 0.180, *p* < 0.001). Among all categories, only nutrition showed an indirect effect. Higher nutrition knowledge had a negative impact on unhealthy eating habits via attitudes towards nutrition (β = −0.003, *p* < 0.05).

## Discussion

This study aimed to determine the relationship between knowledge, attitudes and health risk behaviours among university students. It showed that most university students engage in behaviours that put their health at risk, have poor knowledge about health and have negative attitudes towards risky behaviours. These outcomes demonstrate that despite national efforts to promote health in school and academic contexts, university students commonly practise and accept unhealthy behaviours, even if they have been informed about the harmfulness of certain behaviours.

Although most studies show higher use of tobacco and alcohol (30–32), unbalanced eating habits (33) and risky sexual behaviour (34) among male students, this study found no differences in risky behaviour depending on the student’s gender.

The results showed moderate levels of knowledge about tobacco consumption. As previously noted (30), improving knowledge about the effects of smoking is recommended to reduce smoking rates during university. Similarly, knowledge about STIs was moderate, but the scientific literature presents inconsistent results. Some studies have found the same moderate knowledge (14) and others found low knowledge (13,35) in this dimension. Regardless, it is well known that much of this knowledge remains superficial and is informed by misconceptions and myths (13).

The students’ level of knowledge about alcohol, illicit drugs, self-medication, healthy eating and physical activity was low, which is unsurprising as other studies have already shown insufficient knowledge about health (15–17). Regardless of the correlations shown in the results of this study, scientific evidence has already indicated that knowledge alone does not change health risk behaviour. This study demonstrates the importance of continuing to invest in health education programmes that provide information and improve students’ knowledge. Only then are students likely to make informed, conscious and responsible decisions.

The relationship between the knowledge and behaviour variables was significant (H1), showing that higher health knowledge leads to more risky behaviours. That is, knowledge levels about alcohol are higher among students classified as high-risk consumers, and increases in knowledge about illicit drugs contribute to increases in the likelihood of using these types of drugs. The students who reported practising self-medication in the last 12 months had the highest knowledge levels. Although unexpected, Dermota *et al.* (36) have already observed that the students who engaged in risky behaviours also had higher knowledge levels. One possible explanation for these results and a novel contribution of this study is the identification of a positive relationship between risky behaviours and the propensity of students to actively seek information about the potential harm associated with those behaviours. This finding emphasizes the significance of information-seeking behaviour among individuals with risky tendencies, suggesting a complex relationship between risk-taking and the active pursuit of knowledge regarding the consequences of such behaviours.

The analysis of the relationship between the attitudes and behaviours variables (H2) revealed that unfavourable attitudes influence the adoption of risky behaviours. That is, lack of social support, time constraints, lack of motivation and erroneous beliefs are some of the factors identified in the unhealthy practices of university students, as previously demonstrated by Calamidas and Crowell (3). In this sense, it is important to highlight the significance of the ‘lack of time’ barrier, whether for preparing healthy meals or for physical activity, which is mentioned in the literature as one of the main obstacles and which the results of this study corroborate (33). Another example cited in recent investigations is that many university students believe that condoms reduce pleasure during sexual intercourse (35) and that trust in the sexual partner and the stability of the love relationship justify not using condoms (37) – negative attitudes explicitly stated by the students surveyed in this study. It should be added that although attitudes towards the consumption of illicit drugs have not been presented as a predictor variable for this type of use, other studies have confirmed this relationship, showing that university students tend to consume under the influence of their peers (38). Accordingly, health education programmes in higher education should promote positive attitudes among students to change their unhealthy behaviours. Students are likely to engage in healthy and conscious behaviours if they believe in the importance of their actions. This motivation can be further reinforced by the sense of achievement related to successful experiences and positive feedback on specific behaviours.

This study highlights that health knowledge does not impact health attitudes (H3). This challenges the common assumption that increasing knowledge alone will lead to positive health attitudes. By highlighting the limited impact of knowledge on attitudes, the study underscores the importance of designing effective educational interventions that target attitudes alongside knowledge acquisition to promote healthier behaviours among students. When students recognise the value of their actions, they tend to act responsibly and in a health-promoting manner. Positive experiences and feedback related to certain behaviours can further strengthen this motivation.

The findings generally suggest that health knowledge indirectly influences unhealthy behaviours because it affects attitudes towards health (H4). Put more simply, students with higher levels of knowledge were more likely to have unfavourable attitudes towards healthy behaviours, which, in turn, correlated with a higher prevalence of risk behaviours. This is inconsistent with previous studies, where the results showed that health literacy contributes to preventive health behaviours (1) and that risk behaviours and high levels of health knowledge are not correlated (39).

This study’s results, based on the theory of the KAP model, show that a higher level of knowledge positively influences unhealthy behaviours. Thus, although students engage in numerous health risk behaviours, they are informed about their adverse effects. Other factors that motivate and lead to actions must also be identified when analysing the factors associated with risky health behaviours.

In interpreting the results presented, some limitations should be considered. First, although efforts were made to construct a representative sample of students from the university under study, the findings may not be generalisable to all Portuguese university students due to the study’s limited scope. Generalisations of the results should be made with caution. As demonstrated in other studies (40), although this was a cross-sectional study, conducting mediation analyses was appropriate. Second, the data collection instrument had certain limitations that could affect the validity of the data. These limitations include the possibility of social desirability bias or response bias, as some questions were personal and may have elicited embarrassment, leading to underestimation or overestimation of certain behaviours. In addition, reliance on memory can lead to difficulties in accurately recalling past behaviours, as students were asked about activities in specific time frames (e.g. the last 12 months or the last 30 days).

Furthermore, individual perceptions of the presented items may have differed. For example, there may have been bias in distinguishing between different activity levels, even though examples were given for each type. To mitigate these limitations, all students completed the questionnaires under the same conditions, namely in a classroom setting and on paper. However, this approach may have underrepresented students with certain risk behaviours, such as illicit drug use, because they were less likely to attend class. While the results of this study are informative, the level of evidence must be considered low. The methodological literature emphasises setting temporal precedence when investigating mediation. Therefore, these results must be confirmed through longitudinal studies to determine the temporal sequence and potential bidirectionality (e.g. examining information-seeking following risky behaviours).

## Conclusions

The study findings suggest that knowledge about health and attitudes towards health are significant predictors of risky behaviours, with important implications for policy and programme development. To effectively target and mitigate risky behaviours among students, interventions should prioritise enhancing health knowledge and fostering positive attitudes. It is also crucial to consider the mediating role of attitudes towards health when designing interventions, recognizing their influence on behaviour. Regular and large-scale studies are essential for ongoing assessment of students’ knowledge, attitudes and behaviours. This information serves as a valuable guide for improving socio-educational intervention programmes and public policies, allowing them to be more responsive and tailored to address health risk behaviours. In addition, exploring alternative procedures, potential mediating and moderating effects, and the clustering of risk behaviours can contribute to the development of targeted prevention programmes. By identifying behavioural profiles and understanding common influencing factors, policies and programmes can adopt a comprehensive approach. This enables the design of interventions that simultaneously address multiple behaviours, effectively reaching students with similar behavioural profiles, and leading to more impactful prevention initiatives.

## Supplemental Material

sj-docx-1-ped-10.1177_17579759231195561 – Supplemental material for The relationship between health-related knowledge and attitudes and health risk behaviours among Portuguese university students Supplemental material, sj-docx-1-ped-10.1177_17579759231195561 for The relationship between health-related knowledge and attitudes and health risk behaviours among Portuguese university students by Regina F. Alves in Global Health Promotion

sj-docx-2-ped-10.1177_17579759231195561 – Supplemental material for The relationship between health-related knowledge and attitudes and health risk behaviours among Portuguese university students Supplemental material, sj-docx-2-ped-10.1177_17579759231195561 for The relationship between health-related knowledge and attitudes and health risk behaviours among Portuguese university students by Regina F. Alves in Global Health Promotion

## References

[1] Barsell DJ Everhart RS Miadich SA Trujillo MA . Examining health behaviors, health literacy, and self-efficacy in college students with chronic conditions. Am J Health Educ. 2018; 49: 305–311.

[2] Arsandaux J Montagni I Macalli M Bouteloup V Tzourio C Galéra C . Health risk behaviors and self-esteem among college students: systematic review of quantitative studies. Int J Behav Med. 2020; 27: 142–159. 32072455 10.1007/s12529-020-09857-w

[3] Calamidas EG Crowell TL . A content analysis of college students’ health behaviors. Am J Health Educ. 2018; 49: 133–146.

[4] Crawford LA Novak KB Jayasekare RR . Volunteerism, alcohol beliefs, and first-year college students’ drinking behaviors: implications for prevention. J Prim Prev. 2019; 40: 429–448. 31375975 10.1007/s10935-019-00558-z

[5] Arias-Palencia NM Solera-Martínez M Gracia-Marco L Silva P Martínez-Vizcaíno V Cañete-García-Prieto J , et al Levels and patterns of objectively assessed physical activity and compliance with different public health guidelines in university students. PLoS One. 2015; 10: e0141977. 10.1371/journal.pone.0141977 PMC4633238 26536605

[6] Seo EJ Ahn JA Hayman LL Kim CJ . The association between perceived stress and quality of life in university students: the parallel mediating role of depressive symptoms and health-promoting behaviors. Asian Nurs Res (Korean Soc Nurs Sci). 2018; 12: 190–196. 30103040 10.1016/j.anr.2018.08.001

[7] Ellis B . Risky adolescente behavior: an evolutionary perspective. In: Hewlett B (ed.). Adolescent Identity: Evolutionary, Cultural and Developmental Perspectives. New York: Routledge; 2013, pp.23–29.

[8] World Health Organization. Global Status Report on Alcohol and Health 2018. Geneva: World Health Organization; 2018.

[9] George AM Zamboanga BL Millington E Ham LS . Social anxiety and drinking game behaviors among Australian university students. Addict Behav. 2019; 88: 43–47. 30138776 10.1016/j.addbeh.2018.08.007

[10] Bolden J . Associations among attention problems, learning strategies, and hazardous drinking behavior in a college student sample: a pilot study. Subst Abuse. 2019; 13: 1178221819848356. 10.1177/1178221819848356 PMC6540488 31190852

[11] Al-Zurfi BMN Fuad MD Ghazi HF AbdalQader MA Elnajeh M Baobaid MF . Knowledge, attitudes and beliefs related to drugs among Pahang matriculation students in Malaysia. Int J Public Health Res. 2016; 6: 750–756.

[12] Zahedi L Sizemore E Malcolm S Grossniklaus E Nwosu O . Knowledge, attitudes and practices regarding cervical cancer and screening among Haitian health care workers. Int J Environ Res Public Health. 2014; 11: 11541–11552. 25390794 10.3390/ijerph111111541 PMC4245628

[13] Dutt S Manjula M . Sexual knowledge, attitude, behaviors and sources of influences in urban college youth: a study from India. Indian J Soc Psychiatry. 2017; 33: 319–326.

[14] Folasayo A Oluwasegun A Samsudin S Saudi S Osman M Hamat R . Assessing the knowledge level, attitudes, risky behaviors and preventive practices on sexually transmitted diseases among university students as future healthcare providers in the central zone of Malaysia: a cross-sectional study. Int J Environ Res Public Health. 2017; 14: Article 159. 10.3390/ijerph14020159 PMC5334713 28208724

[15] Gyawali S . Knowledge, attitude and practice of self-medication among basic science undergraduate medical students in a medical school in Western Nepal. J Clin Diagn Res. 2015; 9: FC17–FC22. 10.7860/JCDR/2015/16553.6988 PMC4717826 26816912

[16] Abula K Gröpel P Chen K Beckmann J . Does knowledge of physical activity recommendations increase physical activity among Chinese college students? Empirical investigations based on the transtheoretical model. J Sport Health Sci. 2018; 7: 77–82. 30356484 10.1016/j.jshs.2016.10.010 PMC6180551

[17] Mitra AK Imtiaz A Al Ibrahim YA Bulbanat MB Al Mutairi MF Al Musaileem SF . Factors influencing knowledge and practice of self-medication among college students of health and non-health professions. IMC J Med Sci. 2019; 12: 57–68.

[18] Makiabadi E Kaveh MH Mahmoodi MR Asadollahi A Salehi M . Enhancing nutrition-related literacy, knowledge and behavior among university students: a randomized controlled trial. Int J Nutr Sci. 2019; 4: 122–129.

[19] Abiodun O Sotunsa J Ani F Jaiyesimi E . Knowledge of HIV/AIDS and predictors of uptake of HIV counseling and testing among undergraduate students of a privately owned university in Nigeria. BMC Res Notes. 2014; 7: 639. 25217120 10.1186/1756-0500-7-639 PMC4176830

[20] Werch CE Moore MJ Bian H DiClemente CC Ames SC Weiler RM , et al Efficacy of a brief image-based multiple-behavior intervention for college students. Ann Behav Med. 2008; 36: 149–157. 18800217 10.1007/s12160-008-9055-6 PMC2907163

[21] Al-Otaibi HH . The pattern of fruit and vegetable consumption among Saudi university students. Glob J Health Sci. 2014; 6: 155–162. 10.5539/gjhs.v6n2p155 PMC4825231 24576375

[22] Reis M Ramiro L Matos MG Diniz JA . Nationwide survey of contraceptive and sexually transmitted infection knowledge, attitudes and skills of university students in Portugal. Int J Clin Health Psychol. 2013; 13: 127–137.

[23] Chawla N Neighbors C Lewis MA Lee CM Larimer ME . Attitudes and perceived approval of drinking as mediators of the relationship between the importance of religion and alcohol use. J Stud Alcohol Drugs. 2007; 68: 410–418. 17446981 10.15288/jsad.2007.68.410

[24] Pickard AS Jalundhwala YJ Bewsher H Sharp LK Walton SM Schumock GT , et al Lifestyle-related attitudes: do they explain self-rated health and life-satisfaction? Qual Life Res. 2018; 27: 1227–1235. 29302851 10.1007/s11136-017-1774-3

[25] World Health Organization. Advocacy, Communication and Social Mobilization for TB Control: A Guide to Developing Knowledge, Attitude and Practice Surveys. Geneva: World Health Organization; 2008.

[26] Barry AE Chaney BH Stellefson ML Dodd V . Evaluating the psychometric properties of the AUDIT-C among college students. J Subst Use. 2015; 20: 1–5.

[27] World Health Organization. Healthy diet [Internet]. 2020 [cited 2022 March 30]. Available from: https://www.who.int/news-room/fact-sheets/detail/healthy-diet

[28] Godin G Shephard RJ . Godin leisure-time exercise questionnaire. Med Sci Sports Exerc. 1997; 29(6 Suppl): 36–38.

[29] Hayes AF . Partial, conditional, and moderated moderated mediation: quantification, inference, and interpretation. Commun Monogr. 2018; 85: 4–40.

[30] Askarian M Kouchak F Youssef M Romito LM . Comparing tobacco use knowledge, attitudes and practices between engineering students at a public and Islamic Azad University in Shiraz, Iran 2011. Int J Prev Med. 2013; 4: 1154–1161. 24319555 PMC3843302

[31] Granja GL Lacerda-Santos JT Brilhante DDM Nóbrega ÍD Granville-Garcia AF Caldas Junior AD , et al Smoking and alcohol consumption among university students of the healthcare area. J Public Health (Bangkok). 2020; 28: 45–52.

[32] Balasch M Faucha M Antelo VS Pires CV Carvalho H . Sex-related differences in heavy episodic drinking among young adults living in Porto, Bologna and Tarragona: patterns, protective behaviors and negative consequences [Internet]. J Alcohol Drug Educ. 2018 [cited 2020 April 14]; 62: 72–93. Available from: https://eric.ed.gov/?id=EJ1202787

[33] Hilger J Loerbroks A Diehl K . Eating behaviour of university students in Germany: dietary intake, barriers to healthy eating and changes in eating behaviour since the time of matriculation. Appetite. 2017; 109: 100–107. 27864073 10.1016/j.appet.2016.11.016

[34] Amare T Yeneabat T Amare Y . A systematic review and meta-analysis of epidemiology of risky sexual behaviors in college and university students in Ethiopia, 2018. J Environ Public Health. 2019; 2019: 4852130. 31015844 10.1155/2019/4852130 PMC6446110

[35] Mukherjee A Gopalakrishnan R Thangadurai P Kuruvilla A Jacob KS . Knowledge and attitudes toward sexual health and common sexual practices among college students - a survey from Vellore, Tamil Nadu, India. Indian J Psychol Med. 2019; 41: 348–356. 31391668 10.4103/IJPSYM.IJPSYM_441_18 PMC6657487

[36] Dermota P Wang J Dey M Gmel G Studer J Mohler-Kuo M . Health literacy and substance use in young Swiss men. Int J Public Health. 2013; 58: 939–948. 23842581 10.1007/s00038-013-0487-9

[37] Pastor Y Rojas-Murcia C . A comparative research of sexual behaviour and risk perception in two cohorts of Spanish university students. Univ Psychol. 2019; 18: 1–14.

[38] Helmer SM Mikolajczyk RT McAlaney J Vriesacker B Van Hal G Akvardar Y , et al Illicit substance use among university students from seven European countries: a comparison of personal and perceived peer use and attitudes towards illicit substance use. Prev Med (Baltim). 2014; 67: 204–209. 10.1016/j.ypmed.2014.07.039 25091880

[39] Cook PA Bellis MA . Knowing the risk: relationships between risk behaviour and health knowledge. Public Health. 2001; 115: 54–61. 11402353 10.1038/sj/ph/1900728

[40] Romeo AV Edney SM Plotnikoff RC Olds T Vandelanotte C Ryan J , et al Examining social-cognitive theory constructs as mediators of behaviour change in the active team smartphone physical activity program: a mediation analysis. BMC Public Health. 2021; 21: Article 88. 10.1186/s12889-020-10100-0 PMC7792171 33413209

