# Supplementary material for: The relationship between health-related knowledge and attitudes and health risk behaviours among Portuguese university students
Source: Glob Health Promot. 2023 Sep 16;31(1):36–44. doi: 10.1177/17579759231195561 (PMC11015703; doi:10.1177/17579759231195561)
Supplement: sj-docx-1-ped-10.1177_17579759231195561 – Supplemental material for The relationship between health-related knowledge and attitudes and health risk behaviours among Portuguese university students [file sj-docx-1-ped-10.1177_17579759231195561.docx]

*Table 1*

Descriptive statistics, internal consistencies, and correlations of variables.

|  |  |  |  |  |  |  |  |  |  |  |  |  |  |  |  |  |  |
| --- | --- | --- | --- | --- | --- | --- | --- | --- | --- | --- | --- | --- | --- | --- | --- | --- | --- |
|  | *M*(SD) | α | 1 | 1a | 1b | 1c | 1d | 1e | 1f | 1g | 2 | 2a | 2b | 2c | 2d | 2e | 2f |
| 1. **Health related knowledge**  **(range 1-36)** | **17.77(4.59)** | **.830** |  |  |  |  |  |  |  |  |  |  |  |  |  |  |  |
| *1a. Alcohol Knowledge (range 1-6)* | 2.10(1.14) | .509 | .574^**^ |  |  |  |  |  |  |  |  |  |  |  |  |  |  |
| *1b. Tobacco Knowledge (range 1-6)* | 3.11(1.26) | .556 | .638^**^ | .279^**^ |  |  |  |  |  |  |  |  |  |  |  |  |  |
| *1c. Nutrition Knowledge (range 1-6)* | 2.58(1.31) | .515 | .632^**^ | .232^**^ | .274^**^ |  |  |  |  |  |  |  |  |  |  |  |  |
| *1d. Physical Activity Knowledge (range 1-6)* | 2.74(0.90) | .496 | .492^**^ | .206^**^ | .162^**^ | .236^**^ |  |  |  |  |  |  |  |  |  |  |  |
| *1e. Sexual Risk Knowledge (range 1-6)* | 3.99(1.19) | .573 | .594^**^ | .192^**^ | .265^**^ | .198^**^ | .185^**^ |  |  |  |  |  |  |  |  |  |  |
| *1f. Illicit Drugs Knowledge (range 1-3)* | 1.63(0.96) | .491 | .629^**^ | .228^**^ | .299^**^ | .240^**^ | .188^**^ | .307^**^ |  |  |  |  |  |  |  |  |  |
| *1g. Medication Knowledge (range 1-3)* | 1.60(0.94) | .488 | .592^**^ | .180^**^ | .262^**^ | .266^**^ | .179^**^ | .274^**^ | .405^**^ |  |  |  |  |  |  |  |  |
| **2. Attitudes towards health**  **(range 1-5)** | **2.61(0.48)** | **.800** | **.083^*^** | .097^**^ | .074^*^ | .010 | -.021 | .062 | .123^**^ | -.020 |  |  |  |  |  |  |  |
| *2a. Alcohol Attitudes* | 3.14(0.84) | .762 | .162^**^ | .211^**^ | .124^**^ | .033 | -.026 | .064 | .194^**^ | .043 | .659^**^ |  |  |  |  |  |  |
| *2b. Tobacco Attitudes* | 1.85(0.79) | .749 | .021 | .118^**^ | .003 | .025 | -.017 | -.046 | .101^**^ | -.082^*^ | .623^**^ | .435^**^ |  |  |  |  |  |
| *2c. Nutrition Attitudes* | 2.62(0.87) | .671 | -.069 | -.011 | -0.19 | -.091^**^ | -.015 | -.001 | -.052 | -.085^*^ | .597^**^ | .169^**^ | .194^**^ |  |  |  |  |
| *2d. Physical Activity Attitudes* | 2.26(0.84) | .742 | .017 | -.024 | .004 | .020 | .025 | .020 | .037 | .009 | .643^**^ | .111^**^ | .202^**^ | .370^**^ |  |  |  |
| *2e. Illicit Drugs Attitudes* | 3.66(0.78) | .765 | .136^**^ | -.007 | .144^**^ | .068 | -.003 | .147^**^ | .083^*^ | .079^*^ | .421^**^ | .257^**^ | .098^**^ | .081^*^ | .124^**^ |  |  |
| *2f. Medication Attitudes* | 2.17(0.95) | .708 | -026 | -.015 | -.016 | .019 | .005 | -.040 | -.009 | -.074^*^ | .362^**^ | .098^**^ | .175^**^ | .101^**^ | .173^**^ | .065 |  |
| **3. Health risk behavior**  **(range 1-7)** | **3.88(1.45)** | - | **.146^**^** | .162^**^ | .103^*^ | .076 | .056 | .010 | .197^**^ | -.018 | **.293^**^** | .337^**^ | .340^**^ | .052 | .135^**^ | .028 | .064 |

^*^ *p* < .05; ^**^ *p* < .01
