# Supplementary material for: The relationship between health-related knowledge and attitudes and health risk behaviours among Portuguese university students
Source: Glob Health Promot. 2023 Sep 16;31(1):36–44. doi: 10.1177/17579759231195561 (PMC11015703; doi:10.1177/17579759231195561)
Supplement: sj-docx-2-ped-10.1177_17579759231195561 – Supplemental material for The relationship between health-related knowledge and attitudes and health risk behaviours among Portuguese university students [file sj-docx-2-ped-10.1177_17579759231195561.docx]

*Table 2*

Total, direct and indirect effects of health-related knowledge on health risk behaviors

|  | Total effect  c | Direct effect  c’ | Path a | Path b | Indirect Effects  a*b | | | |
| --- | --- | --- | --- | --- | --- | --- | --- | --- |
|  | *β* | *β* | *β* | *β* | *β* | SD | *t* | CI 95% |
| ***Health-related Knowledge 🡪 Attitudes toward health 🡪 Health risk Behaviors*** | **0.048^**^** | **0.040^**^** | **0.009** | **0.867**^***^ | **0.008^*^** | **0.003** | **2.174** | **.001 - .017** |
| *Alcohol Knowledge 🡪 Alcohol Attitudes 🡪 Risky Drinking* | 0.095^***^ | 0.060^***^ | 0.115^***^ | 0.227^***^ | 0.035^***^ | 0.007 | 5.307 | 0.022 – 0.048 |
| *Tobacco Knowledge 🡪 tobacco Attitudes 🡪 Being an Active Smoker* | 0.011 | 0.011 | 0.002 | 0.173^***^ | 4.641e-4 | 0.004 | 0.901 | -0.017 – 0.008 |
| *Nutrition Knowledge 🡪 Nutrition Attitudes 🡪 Having unhealthy dietary habits* | -0.011 | -0.008 | -0.061^**^ | 0.053^***^ | -0.003^*^ | 0.001 | -2.357 | -0.006 –  -5.300e-4 |
| *Physical Activity Knowledge 🡪 Physical Activity Attitudes 🡪 Leading a Sedentary Lifestyle* | -0.027 | -0.031 | 0.023 | 0.180^***^ | 0.004 | 0.006 | 0.677 | -0.007 – 0.015 |
| *Illicit Drugs Knowledge 🡪 Illicit Drugs Attitudes 🡪 Consuming illicit drugs* | 0.121^***^ | 0.120^***^ | 0.067^*^ | 0.003 | 1.767e-4 | 0.001 | 0.141 | -0.002 – 0.003 |
| *Medication Knowledge 🡪 Medication Attitudes 🡪 Self-medication Practices* | 0.042^*^ | 0.048^*^ | 0.057 | 0.090^***^ | -0.007^*^ | 0.003 | -1.967 | -0.017 –  -2.297e-5 |
